# Low Dose of Apelin-36 Attenuates ER Stress-Associated Apoptosis in Rats with Ischemic Stroke

**DOI:** 10.3389/fneur.2017.00556

**Published:** 2017-10-16

**Authors:** Jian Qiu, Xin Wang, Fei Wu, Lei Wan, Baohua Cheng, Yili Wu, Bo Bai

**Affiliations:** ^1^School of Medicine, Shandong University, Jinan, China; ^2^Institute of Neurobiology, Jining Medical University, Jining, China; ^3^Department of Psychiatry, Jining Medical University, Jining, China; ^4^Shandong Key Laboratory of Behavioral Medicine, Jining Medical University, Jining, China; ^5^Collaborative Innovation Center for Birth Defect Research and Transformation of Shandong Province, Jining Medical University, Jining, China

**Keywords:** apelin-36, ischemic stroke, apoptosis, endoplasmic reticulum stress, unfolded protein response

## Abstract

Cerebral ischemia/reperfusion (I/R) injury-induced cellular apoptosis contributes to neuronal death in ischemic stroke, while endoplasmic reticulum stress (ERS) and subsequently triggered unfolded protein response (UPR) are the major mechanisms of cerebral I/R injury-induced apoptosis. A number of studies indicated that apelin-13 protects neurons from I/R injury-induced apoptosis. Apelin-36, the longest isoform of apelin, has stronger affinity to apelin receptor than apelin-13 does. However, the role of apelin-36 in ischemic stroke is less studied. In addition, preventive administration of apelin was applied in most studies, which could not precisely reflect its therapeutic potential in ischemic stroke. Here, we first reported that low dose of apelin-36, other than apelin-13, administrated after ischemic stroke significantly reduced infarct volume in rats. Moreover, apelin-36 attenuated cerebral I/R injury-induced apoptosis and caspase-3 activation. Furthermore, apelin-36 suppressed I/R injury-induced CHOP and GRP78 elevation, indicating that apelin-36 inhibited ERS/UPR activation. Our study first demonstrated that post-stroke administration of low-dose apelin-36 could attenuate cerebral I/R injury-induced infarct and apoptosis, which is associated with the inhibition of cerebral I/R injury-induced ERS/UPR activation. Our data support the therapeutic potential of apelin-36 in ischemic stroke although further investigation is needed.

## Introduction

Stroke is one of the top leading causes of death and disability worldwide (1). Ischemic stroke, accounting for 87% of stroke cases, contributes to the major portion of death and post-stroke disability (2). The ischemic territory of stroke consists of the core and penumbra, and neuronal apoptosis is the major cause of neuronal death in the area of penumbra (3–5). Endoplasmic reticulum stress (ERS) and subsequent unfolded protein response (UPR) are the major mechanisms in ischemia/reperfusion (I/R) injury-induced cellular apoptosis (5–7). During ERS, 78 kDa glucose-regulated protein (GRP78) is dissociated from two ER membrane proteins, inositol-requiring enzyme 1 (IRE1) and phosphorylation of protein kinase-like ER kinase (PERK), and one Golgi membrane protein, activating transcription factor 6 (ATF6), which subsequently activates three major branches of UPR. Among them, IRE1 and PERK activate the transcription of GRP78 and CCAAT/enhancer binding protein homologous protein (CHOP), while increased expression of GRP78 and CHOP facilitates cell apoptosis (7, 8).

Apelin gene (*APLN)* encodes a 77-amino acid prepro-apelin in human, while the C-terminal 23 amino acids are 100% conserved among human, rat, mouse, and bovine. Prepro-apelin is further cleaved into 13-, 17-, and 36-amino acid peptides from the C-terminus. Apelin receptor (APLNR), belonging to the family of G-protein-coupled receptor, is the endogenous receptor of apelin peptides, which mediates signal transduction *via* G protein (5). Apelin-13 shows the stronger biological activity, while apelin-36 has higher affinity to APLNR (5).

Reduced plasma apelin concentration was detected in patients with myocardial I/R injury (9–11). Moreover, low level of apelin is associated with high incidence of major adverse cardiovascular event post myocardial infarction (12). It suggested that apelin may also play a key role in cerebral I/R injury. A number of evidence indicates that apelin-13 protects neurons and astrocytes from cerebral I/R injury-induced apoptosis in animal models, while only one study showed that apelin-36 had protective effect on cerebral I/R injury-induced apoptosis (13–20). Importantly, preventive administration of apelin-13 and apelin-36 was applied in most studies, which only demonstrated that apelin-13 or apelin-36 have preventive effect on I/R injury-induced apoptosis (13, 14, 16, 17). However, the therapeutic effect of apelin-13 and apelin-36 on ischemic stroke remains elusive. A recent study showed that apelin-13 inhibited I/R injury-induced CHOP and GRP78 elevation in heart, protecting cells from I/R injury-induced apoptosis (21). However, the effect of apelin-36 on cerebral I/R injury-induced CHOP and GRP78 alteration has not be explored.

Accumulated evidence suggests that low dose of apelin-36 may have therapeutic effect on ischemic stroke; meanwhile, it may have less side effect. First, apelin-36 has higher affinity to APLNR compared with apelin-13, suggesting that apelin-36 may be sufficient to maintain APLNR activation at low dose and have relatively long-time effect on APLNR activation compared with apelin-13 (5). In addition, Gu et al. showed that preventive administration of low dose of apelin-36 had protective effects in mice with ischemic stroke, suggesting that low dose of apelin-36 may also have therapeutic effect on ischemic stroke (14). Moreover, it is known that apelin is involved in various processes and functions, which affects drinking behavior, food intake, body weight, blood pressure, body fluid homeostasis, and carcinogenesis, suggesting that low dose of apelin-36 may have less or no side effect for clinical application (22–27). Therefore, it is essential to examine the therapeutic effect of low dose of apelin-36 on ischemic stroke.

In this study, we first reported that low dose of apelin-36, other than apelin-13, administrated after ischemic stroke significantly reduced infarct volume in rats. Moreover, apelin-36 attenuated cerebral I/R injury-induced apoptosis and caspase-3 activation. Furthermore, apelin-36 inhibited I/R injury-induced CHOP and GRP78 elevation. Our study first demonstrated that post-stroke administration of low-dose apelin-36 could attenuate cerebral I/R injury-induced infarct and apoptosis, which is associated with the inhibition of cerebral I/R injury-induced ERS/UPR activation. Our data support the therapeutic potential of apelin-36 in ischemic stroke although further investigation is needed.

## Materials and Methods

### Animals

Adult male Wistar rats (200 ± 20 g) were obtained from Pengyue experimental animal Ltd. (Jinan, China). Rats were housed with free access to food and water under constant temperature (23 ± 2°C) and controlled light conditions (12 h light/dark cycle). The rats were involved in experimental procedures after 5 days of acclimatization. All animal care and procedures described herein were approved by the Animal Care and Use Committee of Jining Medical University, and they were carried out in strict accordance with the guidelines of the Animal Care and Use Committee of Jining Medical University.

### Middle Cerebral Artery Occlusion (MCAO)

The adult rats were anesthetized with 10% chloral hydrate (300 mg/kg, i.p.) and subjected to MCAO using a 2.5 nylon mono-filament (Sigma-Aldrich, USA). The suture was advanced through the common carotid artery into the lumen of the internal carotid artery and advanced 20–25 mm as the distance from the bifurcation until it blocked the origin of right middle cerebral artery as we described previously (16, 17). In the sham operated group, the suture was introduced only into the common carotid artery but not advanced. The neurological function was evaluated at 2 h after MCAO procedure according to the Zea Longa five-point scale: 0 = no appreciable neurological deficits, 1 = failure to extend the contralateral forepaw fully, 2 = circling to the contralateral, 3 = falling to the contralateral, and 4 = failure to walk spontaneously and decreased level of consciousness. After occlusion for 2 h, reperfusion was established by withdrawal of the tip until the suture cleared out of the lumen of the common carotid artery. Body temperature was continuously maintained at 37.0 ± 0.5°C during the surgery using a rat thermostat bench (Taimeng, China).

### Intracerebroventricular (ICV) Injection

After an overnight fast, the rats were anesthetized with an intraperitoneal injection of 10% chloral hydrate (300 mg/kg). A burr hole for ICV administration was carefully made in the skull at 0.8 mm dorsal and 1.6 mm lateral to the right from the bregma using a dremel drill. Total 10 µl apelin-13 (0.03 µg/µl), 10 µl apelin-36 (0.05 µg/µl) (Phoenix Pharmaceuticals, Belmont, CA, USA) (dissolved in aseptic PBS) or 10 µl vehicle only (PBS) were administered into the right lateral ventricle 2 h after MCAO, using a 10 µl microsyringe at the following stereotactic coordinates (AP: −0.8 mm; ML: 1.6 mm; DV: −3.8 mm) as we described previously (16, 17). The experiments were not performed in a blinded manner.

### Estimation of Infarct Volume by Triphenyltetrazolium Chloride (TTC) Staining

Following the MCAO procedure, rats were euthanized at 24 h after reperfusion and the brain were frozen for 30 min at −20°C. The forebrains were dissected into 2 mm coronal slices. Then, slices were incubated with 2% solution of 2, 3, 5-triphenyltetrazolium chloride (TTC, Sigma-Aldrich, USA) in PBS at 37°C for 10 min and then fixed in 4% paraformaldehyde at room temperature. The infarct tissue was illustrated by the complete loss of TTC staining, contrasting with the red-stained viable tissue. Digital photographs were taken for the image analysis. The area of infarction was determined by Image-Pro Plus 6.0 software (Media Cybernetics Inc., Bethesda, MD, USA) as we described previously (16, 17). The experiments were not performed in a blinded manner.

### Terminal Deoxynucleotidyltransferase-Mediated DUTP-Biotin Nick End Labeling (TUNEL)

Terminal deoxynucleotidyltransferase-mediated DUTP-biotin nick end labeling staining was performed following the manufacturer’s instructions (Promega Corporation, Madison, WI, USA) as described previously. Briefly, the forebrains were dissected into 5 µm coronal slices, which were fixed with 4% paraformaldehyde for 30 min and permeabilized with 0.3% triton X-100 for 10 min. After 10 min equilibration, the slices were incubated with rTdT Reaction Buffer and 2× SSC for 15 min to stop rTdT reaction, respectively. The nuclei were counterstained with 10 µg/ml DAPI TUNEL and DAPI stained cells were quantified by counting cells in five non-overlapping fields captured under an Olympus microscope at 20 × 10, and the percentages of TUNEL-positive cells against total cells were calculated. The experiments were not performed in a blinded manner.

### Western Blot Analysis

Brain tissues were homogenized in the RIPA-DOC buffer supplemented with PMSF and phosphatase inhibitor (Roche). The lysed samples were centrifuged at 12,000 for 30 min at 4°C. The supernatants were used for Western blot analysis. The lysates were resolved on 8–12% sodium dodecyl sulfate-polyacrylamide gel electrophoresis and transferred to PVDF membrane at 4°C. The membranes were blocked with 5% skim milk in TBST for 4 h at room temperature and then incubated overnight at 4°C with the following primary antibodies: anti-cleaved caspase-3 (3:5,000, Wanlei), anti-GRP78 (1:1,000; Cell Signaling), anti-CHOP (3:5,000; Wanlei), and anti-β-actin (1:2,000; Zhongshan Golden Bridge Biotechnology). Then, the membranes were incubated with horseradish peroxidase-conjugated anti-rabbit IgG or anti-mouse IgG secondary antibodies (1:5,000, Zhongshan Golden Bridge Biotechnology) for 1.5 h at room temperature or 3 h at 4°C, followed by chemiluminescence assay. The optical density was quantified by Image-Pro Plus VERSion 6.0 (Media Cybernetics Inc., Bethesda, MD, USA). The experiments were not performed in a blinded manner.

### Semi-Quantitative RT-PCR

RNA was isolated from cells using TRI-Reagent (Sigma-Aldrich). Thermoscript Reverse Transcription kit (Invitrogen) was used to synthesize the first-strand cDNA from an equal amount of RNA following the manufacturer’s instruction. The newly synthesized cDNA templates were further amplified in a 10 µL reaction. The following primers were used to specifically amplify mouse *CHOP, GRP78*, and β*-actin* genes: *CHOP* forward 5’- and reverse 5′-; *GRP78* forward 5′- and reverse 5′-; β*-actin* forward 5′- and reverse 5′-. The samples were resolved and analyzed on a 1.5% agarose gel. The experiments were not performed in a blinded manner.

### Statistical Analysis

Values represented mean ± SEM. One-way ANOVA followed by Tukey test was performed by using GraphPad Prism. *p*<0.05 was considered as statistically significant.

## Results

### Low Dose of Apelin-36, but not Apelin-13, Reduces Brain Infarct Volume in Rats with Ischemic Stroke

**Figure 1:**
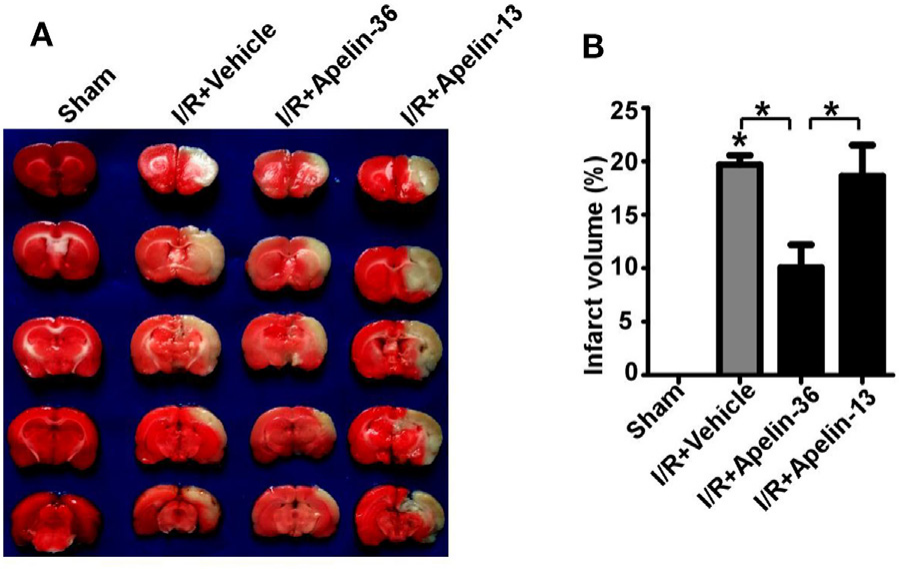
Low dose of apelin-36, but not apelin-13, reduces infarct volume in rats with ischemic stroke. **(A)** The rats were subjected to a sham procedure or 2 h middle cerebral artery occlusion (MCAO) followed by 24 h of reperfusion (I/R). Apelin-36 or vehicle was administrated at 2 h after MCAO procedure. The brain was freshly cut into 2 mm slices and subjected to triphenyltetrazolium chloride (TTC) staining. The infarct tissue was illustrated by the complete loss of TTC staining (white color) and the viable tissue was stained in red color. **(B)** Quantification of infarct volume. Values represent mean ± SEM. *N* = 5, *p < 0.05 by ANOVA followed by Tukey test.

The rat model of cerebral I/R injury was established by 2 h MCAO followed by 24 h reperfusion. Apelin-36 and apelin-13 were administrated after 2 h MCAO by ICV injection, respectively. The infarct area was examined by TTC staining. Cerebral I/R injury dramatically caused infarct in rat brains compared with sham treatment, 19.69 ± 0.88% vs. 0 (*p* = 0.000007). Low dose of apelin-36 significantly reduced infarct volumes compared with vehicle treatment, 10.05 ± 2.15 vs. 19.69 ± 0.88% (*p* = 0.003107) (Figures 1A,B). However, apelin-13 had no effect on reducing infarct volume compared with vehicle treatment, 18.64 ± 2.88 vs. 19.69 ± 0.88% (*p* = 0.696817) (Figures 1A,B). It indicated that low dose of apelin-36, other than apelin-13, administrated after stroke had significant effect on protecting brain from cerebral I/R injury-induced infarct.

### Low Dose of Apelin-36 Attenuates Cerebral I/R Injury-Induced Apoptosis in Rats

**Figure 2:**
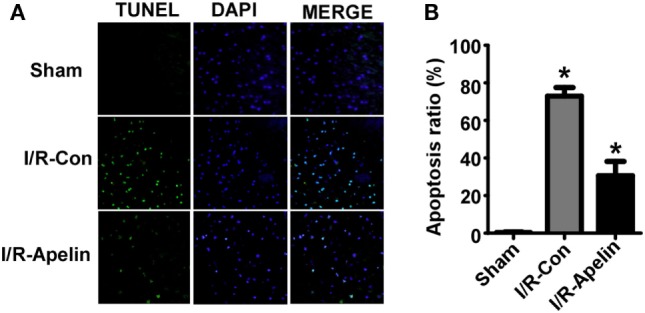
Low dose of apelin-36 attenuates cerebral I/R injury-induced apoptosis in rats. **(A)** The forebrains of rats were dissected into 5 µm coronal slices and terminal deoxynucleotidyltransferase-mediated DUTP-biotin nick end labeling (TUNEL) assay was performed. TUNEL-positive cells are indicated by green color and the nuclei were counterstained with DAPI (blue). **(B)** Quantification of TUNEL assay. Apoptosis ratio was calculated by dividing the number of apoptotic cells by the total cell number. Apelin represents apelin-36. Values represent mean ± SEM. *N* = 5, *p < 0.05 by ANOVA followed by Tukey test.

To determine whether reduced penumbra apoptosis is involved in apelin-36’s protective effect on I/R injury, the neuronal apoptosis was examined in brains of rats with ischemic stroke. Cerebral I/R injury significantly increased cellular apoptosis in rat brains compared with sham treatment, 72.97 ± 4.58% vs. 1.05 ± 0.13% (*p* = 0.000004). Low dose of apelin-36 dramatically reduced I/R injury-induced apoptosis to 30.66 ± 7.56% (*p* = 0.000240) (Figures 2A,B). It indicated that low dose of apelin-36 could protect brain from cerebral I/R injury-induced apoptosis.

### Low Dose of Apelin-36 Attenuates Cerebral I/R Injury-Induced Caspase-3 Activation in Rats

**Figure 3:**
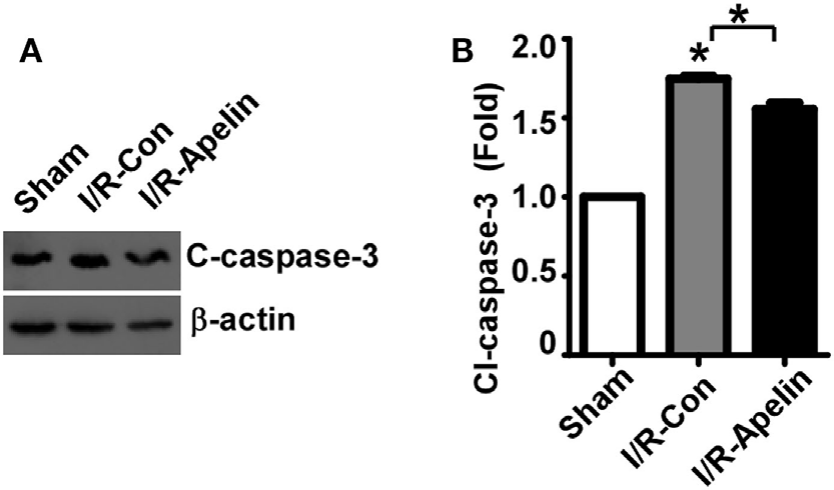
Low dose of apelin-36 attenuates cerebral I/R injury-induced caspase-3 activation in rats. **(A)** The MCA territory of sham treated rats, I/R model rats and I/R model rats treated with apelin-36 (labeled I/R-Apelin) were lysed in RIPA-DOC buffer. Cell lysates were resolved on 12% Tris–Glycine sodium dodecyl sulfate-polyacrylamide gel electrophoresis. Cleaved caspase-3 was detected by cleaved caspase-3 antibody and β-actin served as an internal control was detected by β-actin antibody. **(B)** Quantification of cleaved caspase-3 expression. The ratio of cleaved caspase-3 to β-actin was further normalized to the sham treated rats. Values represent mean ± SEM. *N* = 5, *p < 0.05 by ANOVA followed by Tukey test.

To determine the mechanism of apelin-36’s effect on apoptosis, the alteration of cleaved caspase-3, the active form of caspase-3, was examined. Cerebral I/R injury markedly increased the level of cleaved caspase-3 to 1.75 ± 0.02 fold (*p* < 0.000001) (Figures 3A,B), while low dose of apelin-36 significantly alleviated I/R injury-induced increase of cleaved caspase-3 to 1.56 ± 0.04 fold (=0.000457) (Figures 3A,B). It indicated that low dose of apelin-36 administration could protect brain from cerebral I/R injury-induced apoptosis by inhibiting caspase-3 activation.

### Low Dose of Apelin-36 Inhibits Cerebral I/R Injury-Induced GRP78 and CHOP Elevation in Rats

**Figure 4:**
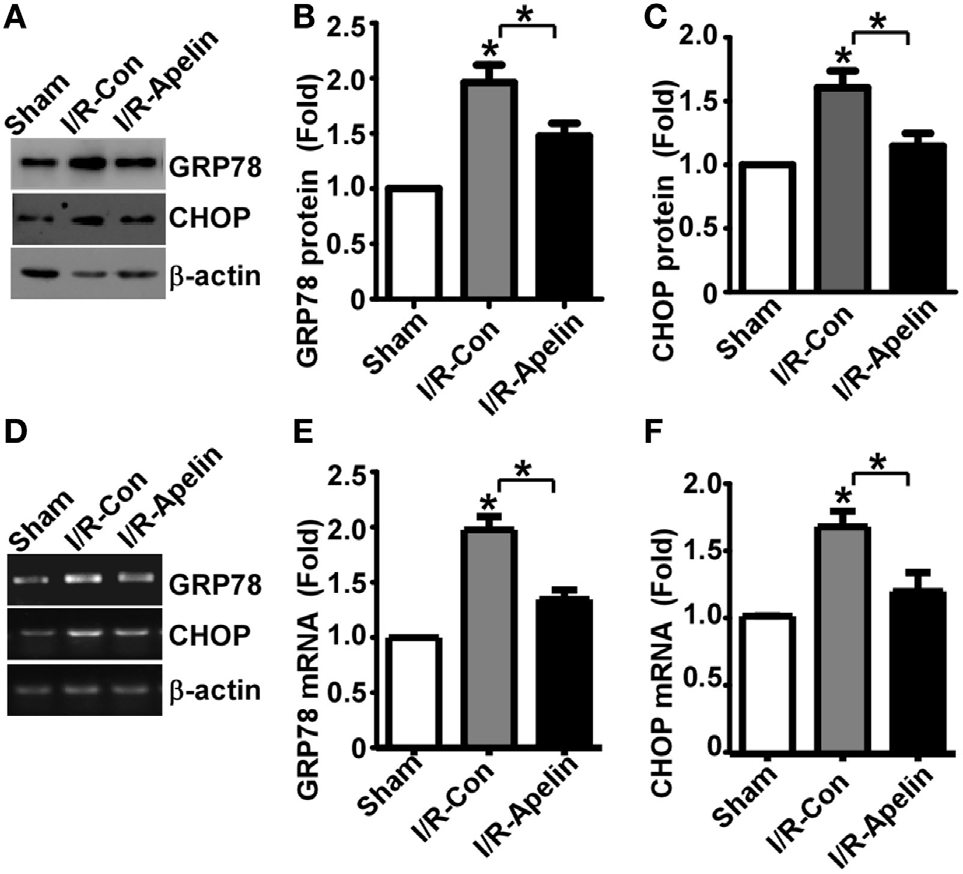
Low dose of apelin-36 inhibits cerebral I/R injury-induced GRP78 and CHOP elevation in rats. **(A)** Rats were subjected to vehicle or apelin-36 treatment at 2 h after middle cerebral artery occlusion (MCAO) procedure. The brain lysates of MCA territory were resolved on 8–12% Tris–Glycine sodium dodecyl sulfate-polyacrylamide gel electrophoresis. GRP78 and CHOP were detected by GRP78 and CHOP antibodies, respectively. β-actin served as an internal control was detected by β-actin antibody. **(B)** and **(C)** Quantification of CHOP and GRP78 protein expression, respectively. Apelin represents apelin-36. **(D)** Rats were subjected to vehicle or apelin-36 treatment at 2 h after MCAO procedure. The mRNA of MCA territory was extracted and RT-PCR was performed. The PCR products of GRP78 and CHOP were resolved on 1.5% agarose gel, respectively. β-actin was served as an internal control. **(E,F)** Quantification of CHOP and GRP78 mRNA expression, respectively. Apelin represents apelin-36. Values represent mean ± SEM. *N* = 5, *p < 0.05 by ANOVA followed by Tukey test.

It is known that ERS and subsequent UPR lead to GRP78 and CHOP elevation, contributing to cellular apoptosis. To investigate whether GRP78 and CHOP alteration is involved in apelin-36’s inhibitory effect on apoptosis, the alteration of GRP78 and CHOP protein was examined. The expression of GRP78 was significantly increased to 1.96 ± 0.16 fold by cerebral I/R injury, *p* = 0.000215 (Figures 4A,B), while apelin-36 markedly reduced I/R injury-induced GRP78 elevation to 1.48 ± 0.12 fold, *p* = 0.000663 (Figures 4A,B). Consistently, the expression of CHOP was significantly increased to 1.63 ± 0.10 fold by cerebral I/R injury, *p* = 0.000127 (Figures 4A,C), while apelin-36 markedly inhibited I/R injury-induced CHOP elevation to 1.13 ± 0.07 fold, *p* = 0.000663 (Figures 4A,C). To further investigate the alteration of GRP78 and CHOP at transcriptional level, mRNA expression of GRP78 and CHOP was examined. GRP78 mRNA was significantly increased to 1.98 ± 0.11 fold by cerebral I/R injury, *p* < 0.000001 (Figures 4D,E), while apelin-36 markedly reduced I/R injury-induced GRP78 elevation to 1.35 ± 0.09 fold, *p* = 0.00095 (Figures 4D,E). Consistently, the mRNA of CHOP was significantly increased to 1.65 ± 0.11 fold by cerebral I/R injury, *p* = 0.0004 (Figures 4D,F), while apelin-36 markedly inhibited I/R injury-induced CHOP elevation to 1.18 ± 0.14 fold, *p* = 0.006 (Figures 4D,F). It indicated that the rescue effect of apelin-36 on apoptosis is associated with its inhibitory effect on the elevation of GRP78 and CHOP in rats with ischemic stroke.

## Discussion

**Figure 5:**
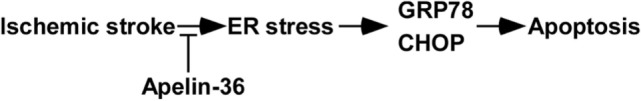
The schematic diagram of apelin-36’s effect on attenuating endoplasmic reticulum stress (ERS)/unfolded protein response activation and apoptosis in ischemic stroke. Low dose of apelin-36 inhibits cerebral I/R injury-induced ER stress and subsequent GRP78 and CHOP elevation, contributing to the reduced apoptosis in ischemic stroke.

Ischemic stroke is a devastating cerebrovascular disease with high morbidity, fatality, and disability rate (2). Protecting against penumbra cell apoptosis is a major strategy to improve post-stroke recovery. ERS/UPR is a major mechanism leading to I/R injury-induced cellular apoptosis in ischemic stroke (5). PERK-CHOP branch and IRE1-GRP78 branch are two major arms of UPR contributing to apoptosis in addition to ATF6 arm. Nakka et al. showed that cerebral I/R injury increased the levels of CHOP and GRP78 mRNA, however, the protein expression of CHOP and GRP78 had not been examined in ischemic stroke (6). In this study, we found that the expression of CHOP and GRP78 was upregulated by cerebral I/R injury, which was associated with cerebral I/R injury-induced infarct and apoptosis in brains of rats (Figure 5). Therefore, inhibiting ERS/UPR-mediated increase of CHOP and GRP78 might be a potential approach to protect I/R injury-induced apoptosis and reduce the infarct volume in ischemic stroke.

A number of studies demonstrated that apelin-13 attenuated neuronal apoptosis in ischemic stroke, facilitating post-stroke recovery (13, 15–17, 19). Compared with apelin-13, apelin-36 has higher affinity to APLNR, suggesting that apelin-36 may have more sustained effect on APLNR activation and even low dose of apelin-36 may be sufficient to maintain APLNR activation (5). However, only one study has been performed to investigate apelin-36’s effect on I/R injury-induced neuronal apoptosis (14). Moreover, no study has compared the differential effect of apelin-13 and apelin-36 on ischemic stroke. In this study, we reported that low dose of apelin-36, approximately 1/550 of regular dose of apelin-13 (0.5 µg/g), significantly reduced infarct volume in rats with ischemic stroke. However, low dose of apelin-13 had no protective effect in rats with ischemic stroke. It highly suggested that low dose of apelin-36, other than apelin-13, has protective effect on ischemic stroke.

Preventive administration of apelin-13 or apelin-36 was applied in the majority of studies of ischemic stroke, which only indicated that apelin-13 or -36 has a preventive effect on protecting brains from I/R injury. However, the therapeutic effect of apelin-13 or -36 on ischemic stroke is less studied. In the current study, we first investigated the therapeutic effect of apelin-36 and apelin-13 on ischemic stroke by administrating low dose of apelin-36 or apelin-13 after ischemic stroke. We found that post-stroke administration of apelin-36 significantly reduced infarct volume in rats. However, apelin-13 could not reduce infarct volume in rats. It suggested that low dose of apelin-36 may have a therapeutic potential for treating ischemic stroke, while the therapeutic effect of apelin-13 on ischemic stroke needs to be further investigated. Moreover, the longer time window of apelin-36 administration should be explored in the future study, which will provide a potential approach to extend therapeutic time window.

A recent study showed that apelin-13 inhibited ERS-induced CHOP and GRP78 elevation in heart, protecting cells from I/R injury-induced apoptosis (21). However, the effect of apelin-36 on ERS-induced CHOP and GRP78 elevation in ischemic stroke has not be explored, particularly the effect of low dose of apelin-36. In this study, we found that low dose of apelin-36 significantly reduced cerebral I/R injury-induced CHOP and GRP78 elevation in rat cortex, indicating that apelin-36 inhibited ERS/UPR activation. Previous studies reported that increased CHOP and GRP78 expression is associated with cellular apoptosis (21, 28–30). It suggested that the inhibitory effect of apelin-36 on cellular apoptosis in ischemic stroke may be mediated by the suppression of ERS/UPR (Figure 5). Moreover, a number of previous studies showed that apelin-13 protects neurons and astrocytes from cerebral I/R injury-induced cellular apoptosis (13–20). We found that apelin-36 significantly reduced cerebral I/R injury-induced cellular apoptosis in the cortex, suggesting that apelin-36 may inhibit both neuronal apoptosis and astrocyte apoptosis in ischemic stroke. Furthermore, apelin-13 attenuates microglia recruitment and activation, contributing to the reduction of inflammatory cytokines and the alleviation of inflammation in ischemic stroke (16, 31). It suggested that apelin-36 may also inhibit cerebral I/R injury-induced inflammation by attenuating microglia recruitment and activation, contributing to the protective effect of apelin-36 on ischemic stroke.

## Ethics Statement

All animal care and procedures described herein were approved by the Animal Care and Use Committee of Jining Medical University, and they were carried out in strict accordance with the guidelines of the Animal Care and Use Committee of Jining Medical University.

## Author Contributions

JQ, BC, YW, and BB conceived and designed the experiments; JQ, XW, FW, LW, and BC performed the experiments and contributed reagents/materials/analysis tools; JQ, YW, and BB wrote the paper. All the authors reviewed the manuscript.

## Conflict of Interest Statement

The authors declare that the research was conducted in the absence of any commercial or financial relationships that could be construed as a potential conflict of interest. The reviewer YC and handling editor declared their shared affiliation.
