# Supplementary material for: Low Dose of Apelin-36 Attenuates ER Stress-Associated Apoptosis in Rats with Ischemic Stroke
Source: Front Neurol. 2017 Oct 16;8:556. doi: 10.3389/fneur.2017.00556 (PMC5650706; doi:10.3389/fneur.2017.00556)

Supplementary data

Figure1

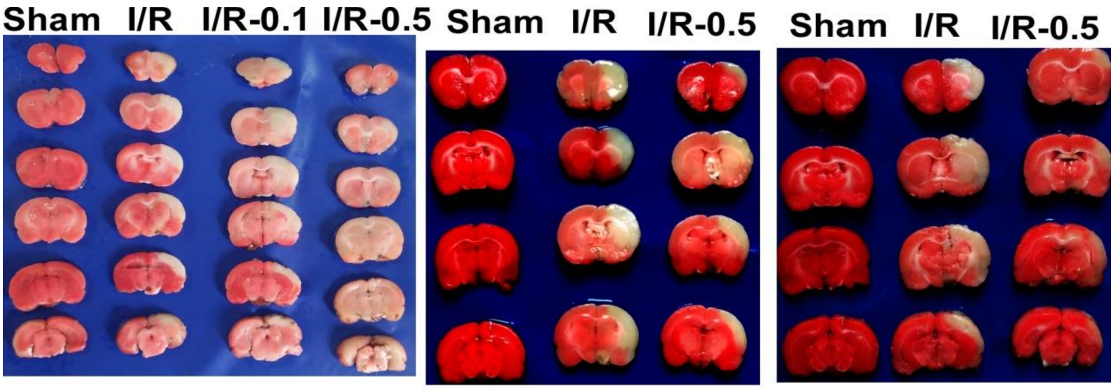

Figure 1

Figure1: The rats were subjected to a sham procedure or 2h MCAO followed by 24 of reperfusion (I/R). 0.1μg (I/R-0.1) and 0.5μg (I/R-0.5) apelin-36 were administrated at 2h after MCAO procedure, respectively. The brain was freshly cut into 2 mm slices and subjected to TTC staining. The infarct tissue was illustrated by the complete loss of TTC staining (white color) and the viable tissue was stained in red color.

Figure 2

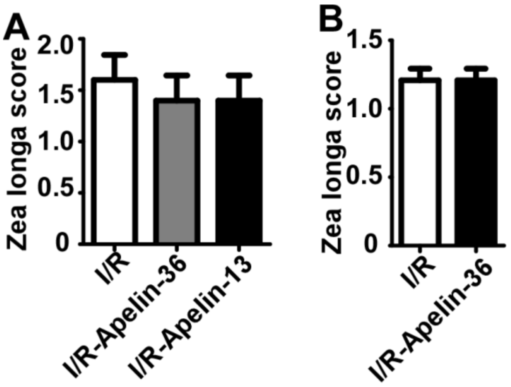

Figure 2

Figure 2: The neurological function was evaluated according to the Zea Longa five-point scale. (A)The Zea Longa scores were evaluated among I/R, I/R-apelin-36 and I/R-apelin-13 groups. N=5. (B) The Zea Longa scores of all rats in the I/R and I/R-apelin-36 groups. N=25.

Figure 3

Full image of western blot in Fig.3 A

Caspase-3 **actin**

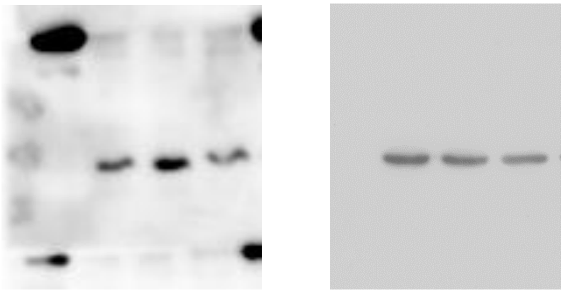

Figure 4

Full image of western blot in Fig.4 A with quantification

GRP78 **CHOP** **actin**

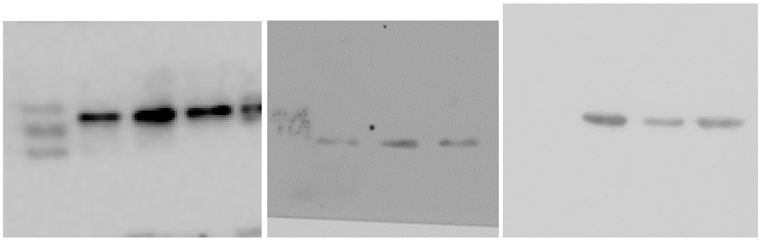

Protein level of GRP78 and CHOP

	GRP78 (mean±SEM, fold)	CHOP (mean±SEM, fold)
**Sham**	1.00	1.00
**I/R-Con**	1.96±0.16	1.63±0.10
**I/R-Apelin**	1.48±0.12	1.13±0.07

Figure 5
RT-PCR in Fig.4 D with quantification

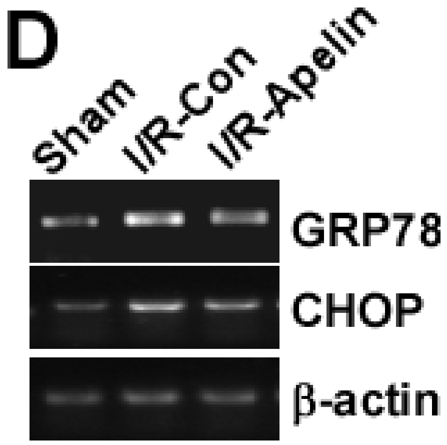

mRNA level of GRP78 and CHOP

	GRP78 (mean±SEM, fold)	CHOP (mean±SEM, fold)
**Sham**	1.00	1.00
**I/R-Con**	1.98±0.11	1.65±0.11
**I/R-Apelin**	1.35±0.09	1.18±0.14

Full image of RT-PCR in Fig.4 D

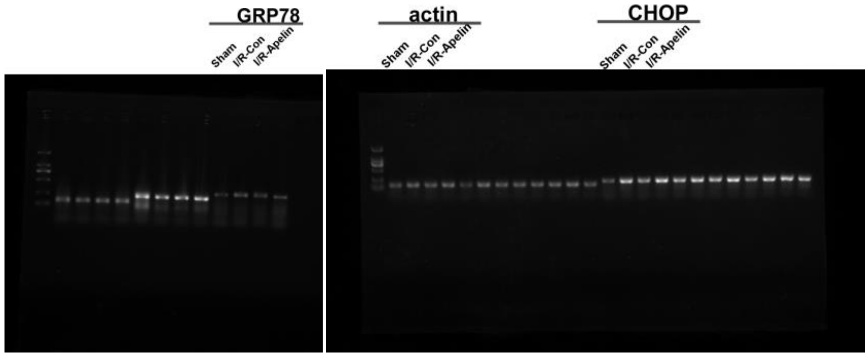

Supplement: Supplementary file 1 [file Presentation_1.PDF]
